# Binaural Pitch Fusion: Effects of Amplitude Modulation

**DOI:** 10.1177/2331216518788972

**Published:** 2018-07-20

**Authors:** Yonghee Oh, Lina A. J. Reiss

**Affiliations:** 1 Department of Otolaryngology, Oregon Health & Science University, Portland, OR, USA

**Keywords:** binaural pitch fusion, amplitude modulation, temporal coherence

## Abstract

Hearing-impaired adults, including both cochlear implant and bilateral hearing aid (HA) users, often exhibit broad binaural pitch fusion, meaning that they fuse dichotically presented tones with large pitch differences between ears. The current study was designed to investigate how binaural pitch fusion can be influenced by amplitude modulation (AM) of the stimuli and whether effects differ with hearing loss. Fusion ranges, the frequency ranges over which binaural pitch fusion occurs, were measured in both normal-hearing (NH) listeners and HA users with various coherent AM rates (2, 4, and 8 Hz); AM depths (20%, 40%, 60%, 80%, and 100%); and interaural AM phase and AM rate differences. The averaged results show that coherent AM increased binaural pitch fusion ranges to about 2 to 4 times wider than those in the unmodulated condition in both NH and bilateral HA subjects. Even shallow temporal envelope fluctuations (20% AM depth) significantly increased fusion ranges in all three coherent AM rate conditions. Incoherent AM introduced through interaural differences in AM phase or AM rate led to smaller increases in binaural pitch fusion range compared with those observed with coherent AM. Significant differences between groups were observed only in the coherent AM conditions. The influence of AM cues on binaural pitch fusion shows that binaural fusion is mediated in part by central processes involved in auditory grouping.

## Introduction

Many studies in normal-hearing (NH) listeners have shown that binaural hearing improves performance in a variety of auditory tasks including sound localization and speech perception in noise. However, recent studies suggest that spectral integration across ears is abnormal in hearing-impaired (HI) listeners (Reiss, Eggleston, Walker, & Oh, 2016). Specifically, binaural pitch fusion, the fusion of dichotically presented tones that evoke different pitches across ears, differs in HI listeners. NH listeners exhibit narrow binaural pitch fusion ranges (0.1–0.2 octaves), that is, only fuse tones with small interaural frequency differences (Odenthal, 1963; Reiss et al., 2017; Thurlow & Bernstein, 1957; Van den Brink, Sintnicolaas, & van Stam, 1976). In contrast, HI listeners can exhibit broad binaural pitch fusion, fusing tones that differ by as much as 3 to 4 octaves (Oh & Reiss, 2017; Reiss et al., 2017). In addition, this broad binaural pitch fusion leads to averaging of different tones (Oh & Reiss, 2017) or even phoneme percepts across the ears such that speech perception is sometimes worse with two ears than with either ear alone (Reiss et al., 2016). HI listeners already have poorer frequency resolution, and broad binaural fusion and the associated spectral averaging and smearing across ears could further reduce a HI listener’s effective frequency resolution, and thus reduce binaural benefits for localization and speech perception in the presence of background noise (Oh et al., 2017).

It should be noted that previous studies of binaural pitch fusion focused on steady-state acoustic or electric signal presentation without any temporally varying components. In reality, most real-world sounds, including speech, contain temporal as well as spectral variation. There is evidence that the information in different frequency regions within ears tends to be fused whenever the temporal envelope matches in frequency and in phase (Bregman, Abramson, & Doehring, 1985). In addition, the results of early studies using tones, noise, and speech (Broadbent & Ladefoged, 1957; Leakey, Sayers, & Cherry, 1958) suggest that coherent variation in the temporal envelope facilitates the fusion of dichotic sounds. Although the mechanism for facilitation of binaural fusion is not yet fully understood, some physiological data suggest that a common rate of neural periodicity could enhance binding regions of the spectrum together into a single perceived sound, possibly via a topographic map of modulation rate tuning (for a review, see Joris, Schreiner, & Rees, 2004).

However, it is important to note that perception of such temporal envelope fluctuations, or amplitude modulation (AM), may differ in listeners with hearing loss. Detection thresholds, or the smallest AM that can be detected, can be better in HI listeners (e.g., Lüscher & Zwislocki, 1949; Moore, Shailer, & Schooneveldt, 1992; Moore, Wojtczak, & Vickers, 1996; Schlittenlacher & Moore, 2016), potentially due to abnormal loudness growth and recruitment.

The goal of the current study was to further investigate how binaural pitch fusion can be influenced by temporal properties of the stimuli, specifically AM of the temporal envelope, and whether effects differ with hearing loss, which affects AM detection and discrimination. This study focused on the effects of systematically varying AM rates, AM depths, and interaural AM phase and AM rate differences on binaural pitch fusion in NH and HI listeners, and comparing the effects between groups.

## Methods

### Subjects

**Figure 1.**
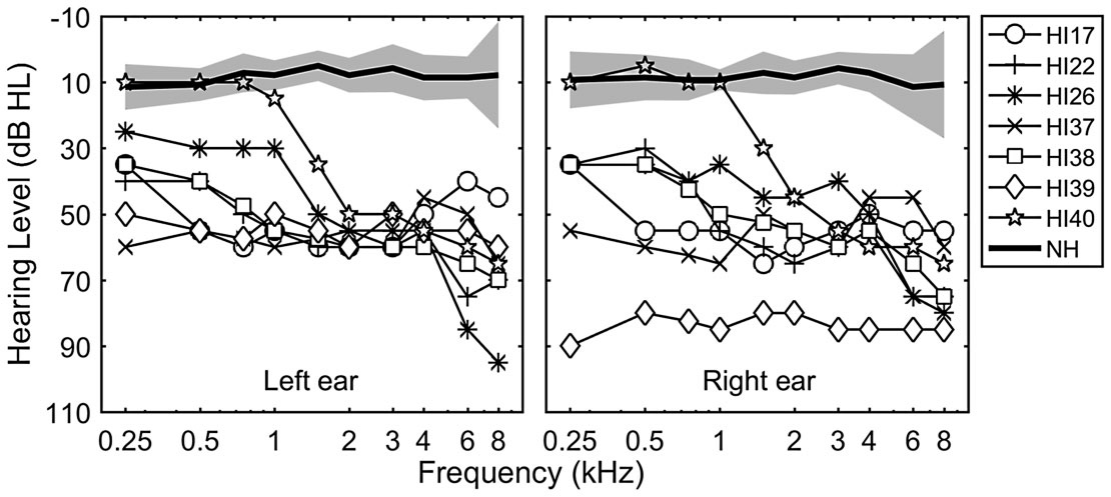
Unaided audiograms for the left ears (left panel) and the right ears (right panel) of the subjects in this study. Thin lines with symbols show individual thresholds for HA users. Thick lines and shaded areas represent averaged thresholds and standard deviations for NH subjects. HI = hearing impaired; NH = normal hearing.

Seven NH adults (ages 22 to 74; 2 males and 5 females) and 7 bilateral hearing aid (HA) users (ages 36 to 77; 3 males and 4 females) participated in this study. NH was defined as air conduction thresholds ≤25 dB hearing level from 250 to 4000 Hz (all testing was conducted with tones below 4000 Hz). Group-averaged audiograms for NH subjects are shown for left and right ears in Figure 1 (see thick solid lines).

All HA users had moderate to severe hearing losses in both ears and relatively symmetric losses between ears, except for subject HI39 who had a 30 dB difference (see individual audiograms in Figure 1). All HA users had at least 1 year of experience with both of their HAs. The detailed HA users’ demographic data including age, gender, duration of moderate–severe hearing loss, duration of HA use, daily hours of HA use, and HA models are shown in Table 1.

**Table 1.** Demographic Information for HA Users: Age, Gender, Duration of Moderate–Severe Hearing Loss, Duration and Daily Hours of HA Use, and HA Model.

Subject ID	Age (years)	Gender	Duration of HL (years)	Duration of HA use (years)	Daily HA use (hr/day)	HA model (ear if only one worn)
HI17	63	F	2	1; 5	10; 0	Oticon Nera (R)
HI22	54	M	6	6	16	Rexton Mosaic BTE
HI26	63	F	23	10	14	Rexton Revera WL
HI37	36	F	15	13	10	Siemens Silk Primax
HI38	72	F	19	19	16	Phonak Audeo V90
HI39	74	M	30	21	10	ReSound Linx
HI40	77	M	7	6	16	Phonak Audeo V90

*Note*. “;” denotes different numbers for left and right ears if the ears are different. HA = hearing aid; HI = hearing impaired; HL = hearing loss.

All subjects were screened for normal cognitive function using the Mini-Mental Status Examination with a minimum score of 25 out of 30 required to qualify (Folstein, Folstein, & McHugh, 1975; Souza, Boike, Witherall, & Tremblay, 2007). Tympanometry was also conducted for all subjects to verify normal middle ear function. Both ethical and methodological approvals were obtained from the Institutional Review Board of Oregon Health and Science University. All subjects provided written informed consent.

### Stimuli and Procedures

All experiments were conducted in a double-walled, sound-attenuated booth. Signals were generated at a sampling rate of 44.1 kHz with MATLAB (version R2010b, MathWorks); processed through an ESI Juli sound card, TDT PA5 digital attenuator, and HB7 headphone buffer; and presented over Sennheiser HD-25 headphones. Each headphone’s frequency response was equalized using calibration measurements obtained with a Brüel & Kjær sound level meter with a 1-inch microphone in an artificial ear.

All stimuli consisted of pure tones with 10-ms raised-cosine onset/offset ramps. Prior to all experiments, loudness balancing was conducted using a method of adjustment. First, 300-ms tones at 0.25, 0.375, 0.5, 0.625, 0.75, 0.875, 1, 1.25, 1.5, 2, 3, and 4 kHz in the reference ear were initialized to “medium loud and comfortable” levels corresponding to a 6 or “most comfortable” on a visual loudness scale from 0 (no sound) to 10 (too loud). Loudness for the comparison ear was then adjusted for each frequency to be equally loud to a tone in the reference ear during sequential presentation across the ears, based on subject feedback. Here, all loudness balancing adjustments were repeated with a fine attenuation resolution (0.1 dB steps for HA users and 0.5 dB steps for NH listeners) until equal loudness was achieved with all comparison sequences within and across ears. The frequencies and order of presentation were randomized to minimize the effect of biases such as time-order error and overestimation of the loudness for high-frequency tones (Florentine, Popper, & Fay, 2011). Interpolation (on a dB scale with a linear frequency) was then used to determine appropriate levels for all tone frequencies used in testing. The detailed comfortable sound levels across subjects and tone frequencies are described in Table 2. This loudness balancing procedure was performed to minimize use of level-difference cues and maximize focus on pitch differences as the decision criteria. Note that only subject HI39 showed asymmetric equal-loudness balanced levels across ears.

**Table 2.** Comfortable Sound Levels Obtained by the Equal-Loudness Balancing for All Subjects.

Subject ID	Ear	Comfortable level by equal-loudness balance (dB SPL)											
		Frequency (kHz)											
		0.25	0.375	0.5	0.625	0.75	0.875	1	1.25	1.5	2	3	4
NH	*M*	75	71	70	69	68	68	70	71	72	70	67	66
	*SD*	2.3	2.3	1.9	1.2	0.5	0.7	0.8	1.1	0.9	1.7	2.0	1.9
HI17	L	84	80	86	85	81	81	82	86	90	90	93	95
	R	87	86	85	85	86	87	92	95	98	98	96	98
HI22	L	96	92	91	91	93	94	94	97	99	102	102	99
	R	91	88	88	86	90	92	92	95	96	102	100	97
HI26	L	80	76	73	71	71	74	77	79	85	88	80	83
	R	81	75	75	76	77	75	71	72	72	76	76	78
HI37	L	94	90	91	91	91	92	92	94	95	98	98	93
	R	93	90	90	91	92	92	92	93	94	98	98	95
HI38	L	83	83	83	84	86	86	87	88	89	90	88	87
	R	82	82	83	83	83	84	85	85	89	90	88	86
HI39	L	89	85	86	81	81	82	82	84	80	78	75	73
	R	121	118	116	115	116	116	113	106	104	102	98	98
HI40	L	74	70	71	71	71	72	72	79	85	88	90	90
	R	73	70	70	71	72	72	70	75	76	85	88	88

*Note*. Only group-averaged levels are shown for NH subjects. HI = hearing impaired; NH = normal hearing; SPL = sound pressure level.

To determine the frequencies at which binaural beat cues were present and would potentially interfere with the fusion task, upper limit frequencies for detection of interaural phase differences (IPDs) were measured by using a rapid, adaptive IPD test (Grose & Mamo, 2010). A three-interval, three-alternative forced choice adaptive procedure was used, in which three binaural tone stimuli were presented at the test frequency, with 800-ms duration. For two of the intervals, stimuli were in phase between ears over the whole duration. For the third interval, the phase was inverted to be out of phase between ears every 200 ms such that listeners sensitive to phase cues at that frequency would effectively hear a binaural beat. Detailed procedures for the IPD test have been described previously in Reiss et al. (2017). For all subjects, the IPD thresholds were lower than all of the reference frequencies (i.e., 2 kHz) used in this study (mean and std = 989 ± 434 Hz for NH subjects and 460 ± 253 Hz for HA users).

For the dichotic fusion range measurement, both reference and dichotic comparison tones were presented simultaneously in a 1,500-ms single interval, in which subjects were asked to indicate whether they heard a single fused sound or two different sounds in each ear. If subjects heard a sound only in one ear (lateralization), they were instructed to indicate that they heard one sound, as lateralization is an indication of fusion. The reference tone frequency for one ear, designated the reference ear, was fixed at 2 kHz. For NH subjects, comparison tone frequencies presented to the contralateral ear were varied in 1/16 octave steps within the range of 1.414 and 2.828 kHz (i.e., 17 frequencies). For HA users, larger step sizes and a broader range of frequencies were used for the comparison tone (i.e., 1/4 octave steps between 0.25 and 4 kHz). The comparison tone frequencies were pseudorandomly varied at each trial, and multiple presentations of the same reference and comparison tone pairs were provided. The reference ear was chosen randomly for each subject. Single sound responses were assigned a value of 1, and two sound responses were assigned a value of 0.

In the unmodulated (control) condition, both reference and comparison tones had a constant envelope. In the coherent AM conditions, the tone envelopes were amplitude modulated with AM rate and AM depth varied across conditions. The AM rates were 2, 4, and 8 Hz, and the AM depths varied between 20% and 100% in steps of 20 percentage points (i.e., 20%, 40%, 60%, 80%, and 100%). These Hresslow AM rates were chosen from the range of 2 to 8 Hz because this range contains prominent envelope fluctuations of the speech signal that contribute significantly to speech intelligibility (Füllgrabe, Stone, & Moore, 2009). In the first incoherent AM condition, IPDs were varied between 45° and 180° in steps of 45° (i.e., 45°, 90°, 135°, and 180°). In the second incoherent AM condition, different AM rates were presented to each ear (i.e., a fixed 2-Hz AM tone in the reference ear and tones with a 2-, 3-, 5-, or 7-Hz AM rate in the comparison ear) with randomization of the starting phases of the modulator envelopes on each trial. The modulation depth was fixed at 60% for both incoherent AM conditions. Fusion range measurements were collected for 24 conditions (1 unmodulated condition, 15 coherently modulated conditions with 3 different AM rates × 5 different AM depths, and 8 incoherently modulated conditions). The results for all experiments were averaged with two separate runs for each condition. All statistical analyses were conducted on octave-scale data in SPSS (version 25, IBM).

## Results

### Binaural Pitch Fusion Ranges and Fusion Centers

**Figure 2.**
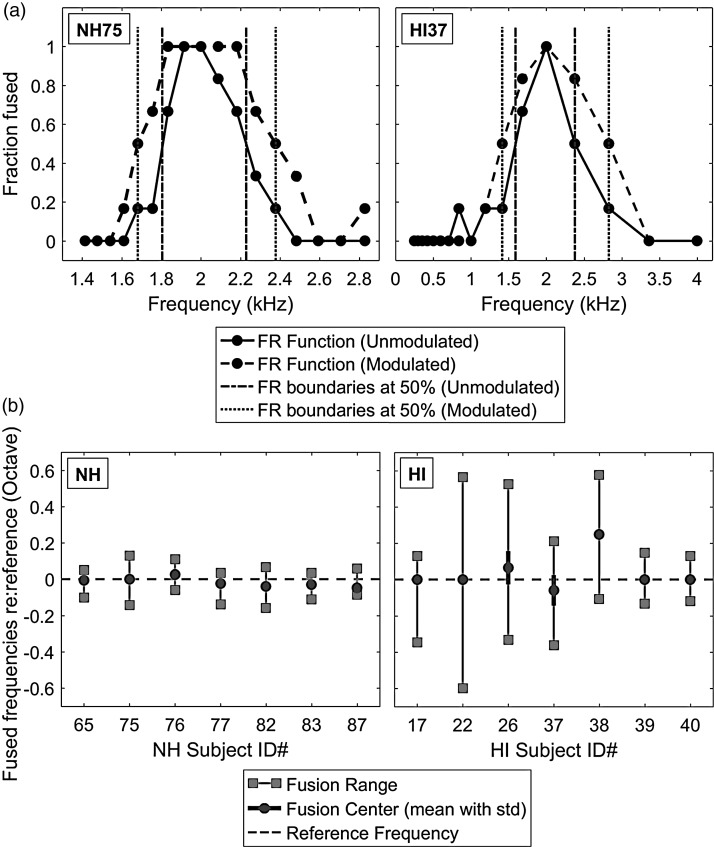
Binaural fusion range and fusion center results. (a) Example fusion functions are shown for representative NH and HA subjects in the left and right panels, respectively. The solid line with filled circles indicates an example fusion function in the unmodulated condition, and the dashed line with filled circles shows an example in the coherently modulated condition with 4-Hz rate and 60% depth. The fusion range is the frequency range of pair tones (in the contralateral ear) that fused with the reference tone (in the reference ear) more than 50% of the time (frequencies between the vertical dashed-dotted lines for the unmodulated condition and frequencies between the vertical dotted lines for the coherently modulated condition; fraction fused >0.5). (b) Summary of fusion range and fusion center results (in octave) for individual NH subjects (left) and HA users (right) in the unmodulated condition. Fusion ranges are indicated by vertical solid lines. Fusion centers are indicated by the filled circles, with the short solid lines showing the standard deviations. HI = hearing impaired; NH = normal hearing.

Fusion functions were computed as the average of subject responses to multiple presentations of each stimulus pair as a function of comparison tone frequency. Values near 0 indicate comparison tone frequencies that did not often fuse with the reference tone (were heard as two sounds), while values near 1 indicate comparison tone frequencies that were often fused with the reference tone (were heard as one sound). Figure 2(a) shows example fusion functions for a representative NH subject (NH75; left panel) and an HA user (HI37; right panel). Vertical dashed-dotted and dotted lines indicate the 50% boundaries for each fusion function in the unmodulated condition (solid lines with filled circles) and the coherently modulated condition with 4-Hz rate and 60% depth (dashed lines with filled circles), respectively. The examples illustrate that each fusion range, defined as the frequency range between these vertical lines, broadened in the coherently modulated condition. A similar fusion range increment with coherent modulation was observed in both subjects (0.27 to 0.55 octaves for NH75 and 0.57 to 1.01 octaves for HI37). Center frequencies of the fusion ranges (i.e., fusion centers) were also used as a measure of the overall frequency offset of the fusion range relative to the reference frequency and were calculated as the weighted average of the frequencies within the fusion range. Center frequencies were not affected by the coherent AM in both subjects (not shown).

Figure 2(b) shows summary fusion ranges (solid vertical lines) and fusion centers (filled circles) in the unmodulated condition, with the left and right panels showing results for NH and HA subjects, respectively. To determine whether hearing loss (NH vs. HA) affected either fusion range or fusion center changes in the unmodulated condition, two-tailed independent-samples *t* tests were performed with Levene’s test for equality of variances. Generally, all NH subjects exhibited significantly narrower fusion ranges than HA subjects (NH: 0.18 ± 0.05 octaves, HA: 0.61 ± 0.32 octaves; *t*(6.262)* = *−3.456, *p* = .013, with unequal variance), which is consistent with the previous study (Reiss et al., 2017). Averaged fusion centers were 0.04 ± 0.1 octaves higher than the reference frequency for NH subjects and 0.02 ± 0.03 octaves lower than the reference frequency for HA subjects; however, differences in fusion centers between NH and HA subjects were not significant (*p* > .1, with equal variance). In addition, separate one-sample *t* tests showed nonsignificant offsets of fusion centers from the reference frequency for both NH and HA subjects (*p* > .1 in both subject groups).

### Effects of Coherent AM on Binaural Pitch Fusion

**Figure 3.**
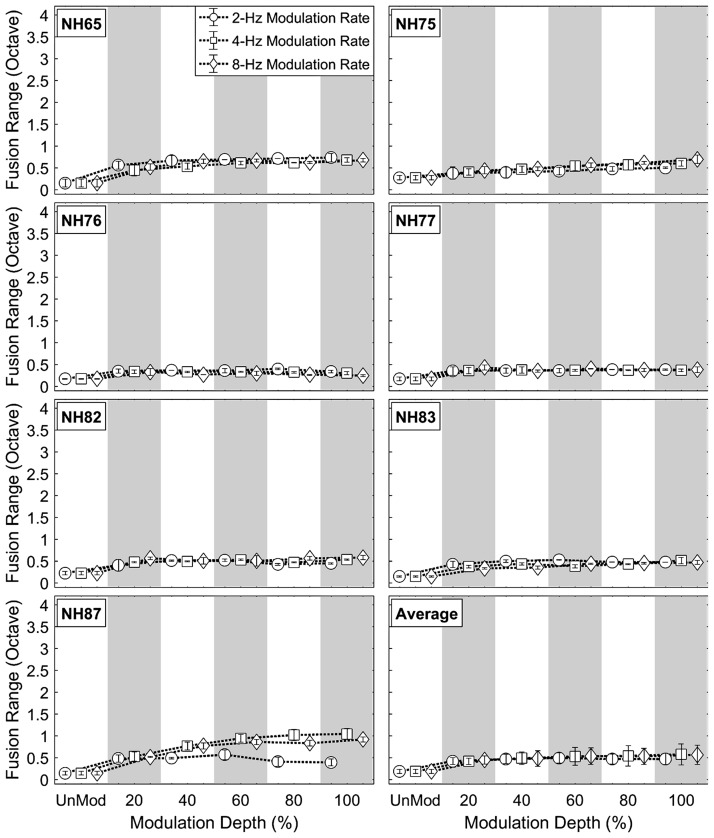
Individual and averaged fusion range results for NH subjects at various coherent amplitude-modulation rates (2, 4, and 8 Hz) and depths (20%, 40%, 60%, 80%, and 100%). The open circle, square, and diamond symbols indicate mean fusion ranges (in octave) at modulation rates of 2, 4, and 8 Hz, respectively. Error bars represent standard deviations around the mean. NH = normal hearing.

**Figure 4.**
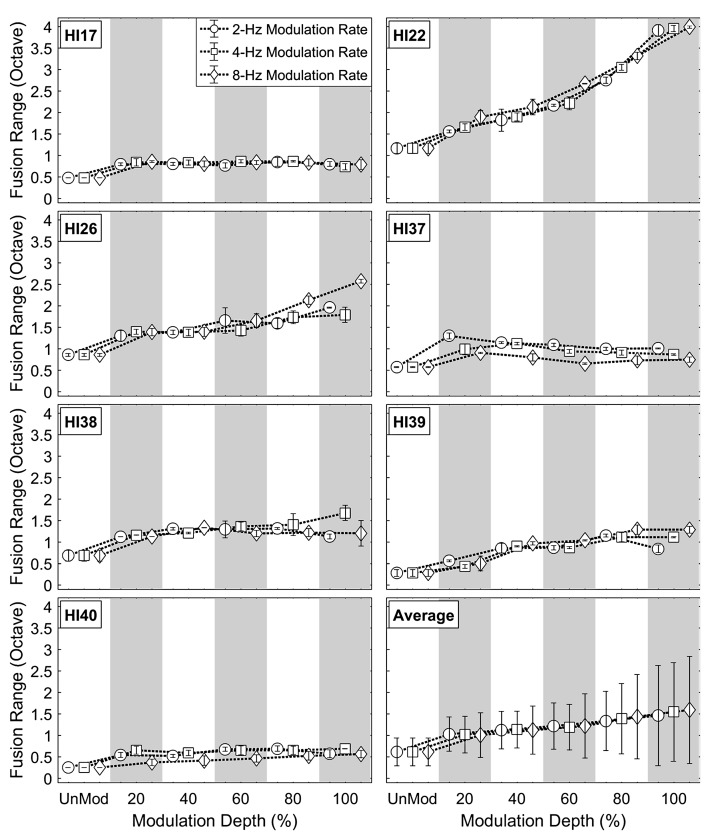
Individual and averaged fusion range results for HA users at various coherent amplitude-modulation conditions. Plotted as Figure 3. HI = hearing impaired.

Figures 3 and 4 show individual and averaged fusion range results at various coherent AM conditions (2, 4, and 8 Hz) as a function of modulation depth (0%, 20%, 40%, 60%, 80%, and 100%) for NH and HA subjects, respectively. In both subject groups, the individual results show that fusion ranges broadened with increased modulation depths at all AM rates tested, to about 2 to 4 times wider than those in the unmodulated condition. Even shallow temporal envelope fluctuations (i.e., the 20% AM depth) increased fusion ranges in most of the AM rate conditions across the subjects.

Individual variation in changes in fusion range with increases in coherent AM are apparent in both NH and HA subject groups. In the majority of the subjects, the fusion ranges asymptoted at various AM depths for all AM rates (see NH65, NH76, NH77, NH82, and NH83 in Figure 3 and HI17, HI38, and HI40 in Figure 4). The maximum fusion range also varied with AM rate. For example, at modulation depths between 40% and 100%, subject NH65 had broader fusion ranges at the 2-Hz AM rate than those at 4 and 8-Hz AM rates. In some subjects, the fusion ranges continued to increase with AM depth for all AM rates (see NH75 in Figure 3 and HI22 and HI26 in Figure 4). In other cases, the fusion ranges showed nonmonotonic changes with modulation depth, depending on AM rate (NH87 in Figure 3 and HI37and HI39 in Figure 4). These nonmonotonic changes were particularly apparent at modulation depths between 20% and 100% in the 2-Hz AM rate condition (circles) for subject NH87 and in the 8-Hz AM rate condition (diamonds) for subject HI37.

Group-averaged data show that coherent AM increased binaural pitch fusion for both subject groups (see bottom right panels in Figures 3 and 4). Here, a three-way repeated-measure analysis of variance (RM-ANOVA) was performed with fusion range as the dependent variable, AM rate and depth as within-subject factors, and listener group (NH vs. HA) as a between-subject factor. Greenhouse-Geisser correction was applied where the assumption of sphericity was violated. The results showed significant main effects of AM depth, *F*(1.131, 13.572) = 11.036, *p* = .004, $\eta_{p}^{2}$^ ^= .479, and listener group, *F*(1, 12) = 31.066, *p* = .013, $\eta_{p}^{2}$^ ^= 0.418, but no main effect of the AM rate and no significant AM Rate × AM Depth interaction (*p* > .3 in both cases). Post hoc pairwise comparisons using Bonferroni correction were performed to better understand the main effect of AM depth. Relative to the unmodulated condition (0% AM depth), relatively shallow temporal fluctuations (20%–40% AM depths) significantly increased binaural fusion ranges at all AM rates tested (2, 4, and 8 Hz; *p* < .0001 in all AM rate conditions).

**Figure 5.**
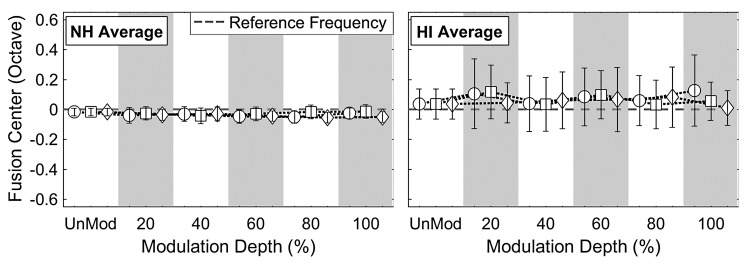
Averaged fusion center results for NH (left) and HA (right) subjects at various coherent modulation rates (2, 4, and 8 Hz) and depths (20%, 40%, 60%, 80%, and 100%). The fusion center was calculated as the average peak frequency of the fusion function (i.e., Figure 2(a)). The open circle, square, and diamond symbols indicate mean fusion centers (in octave) at 2-, 4-, and 8-Hz coherent modulation rates, respectively, along with the standard deviations (overlaid vertical lines). HI = hearing impaired; NH = normal hearing.

Figure 5 shows, on average, minimal effects of AM depth on center frequency of the fusion range (i.e., fusion center) for NH and HA subjects. In both subject groups, the fusion centers in all modulated conditions were slightly shifted from those in the unmodulated condition (NH: −0.04 ± 0.005 octaves, HA: 0.08 ± 0.019 octaves), though RM-ANOVA showed that main effects of both AM rate and depth on the fusion-center shifts were not significant, and there was no significant AM Rate × AM Depth interaction (*p* > .2 in all cases). In addition, no significant main effect of listener group was observed on the fusion-center shifts (*p* > .1).

### Effects of Incoherent AM on Binaural Pitch Fusion

Three subjects in the NH and three subjects in the HA subject groups were also tested on incoherent AM conditions, in which either the AM phase or AM rate differed between ears.

**Figure 6.**
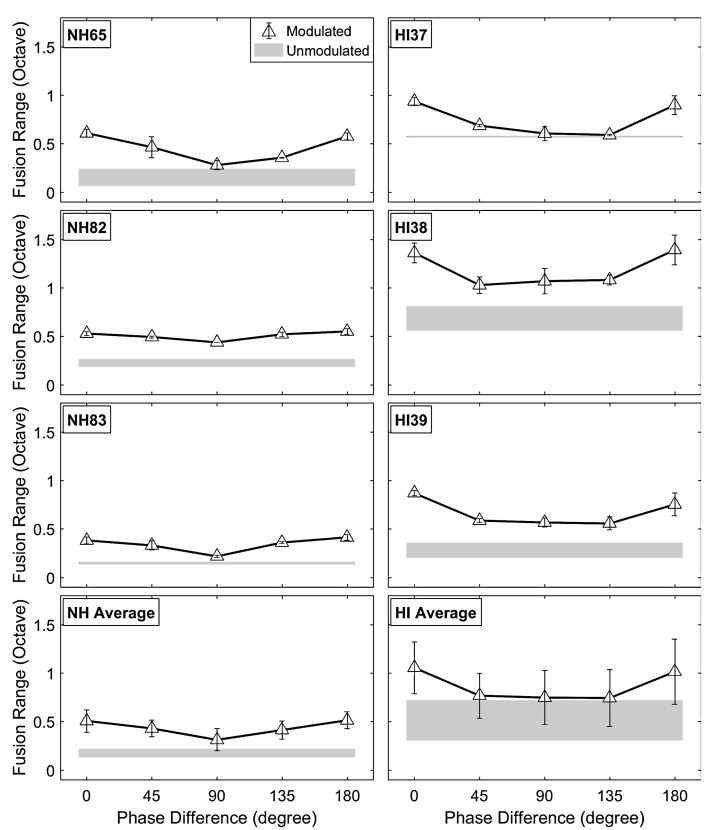
Effects of varying interaural AM phase differences on fusion ranges for representative NH (left) and HA (right) subjects. The bottom panels show the averaged results for both subject groups. The triangle symbol indicates mean fusion ranges (in octave) with phase differences (0%, 25%, 50%, 75%, and 100%) at fixed modulation rates of 4 Hz and depth of 60%. The shaded area represents fusion ranges (including standard deviations) in the unmodulated condition. HI = hearing impaired; NH = normal hearing.

For the first incoherent AM condition, Figure 6 shows the individual and averaged fusion range results as a function of interaural AM phase differences (0°, 45°, 90°, 135°, and 180°) for NH and HA subjects. Note that all stimuli were fixed at the 4-Hz AM rate and 60% AM depth, and only the AM phase difference between two dichotic stimuli was manipulated. In both subject groups, the individual results show that interaural AM phase differences between 45° and 135° decreased fusion ranges compared with the coherent AM condition (0° phase difference) for all subjects; however, the decreased fusion ranges in the 90° phase difference condition were still broader than those observed in the unmodulated conditions (shaded areas). In contrast, the 180° phase difference led to similar fusion ranges compared with those in the coherent AM condition (0° phase difference).

The results from RM-ANOVA with AM phase differences as within-subject factors and listener group as between-subject factor showed a significant main effect of interaural AM phase differences on fusion ranges, *F*(4, 16) = 28.391, *p* < .0001, $\eta_{p}^{2}$^ ^= 0.877, and a marginal main effect of listener group, *F*(1, 4) = 6.564, *p* = .063, $\eta_{p}^{2}$^ ^= 0.62, but no Interaural AM Phase Differences × Listener Group interaction (*p* > .1). Post hoc analyses using Bonferroni correction indicated that the 90° phase difference led to significantly decreased fusion ranges compared with either 0° phase difference (*p* < .023) or 180° phase difference condition (*p* < .026). Results from a separate RM-ANOVA with fusion center as the dependent variable showed no significant main effects of interaural AM phase difference or listener group (*p* > .2 in both cases).

**Figure 7.**
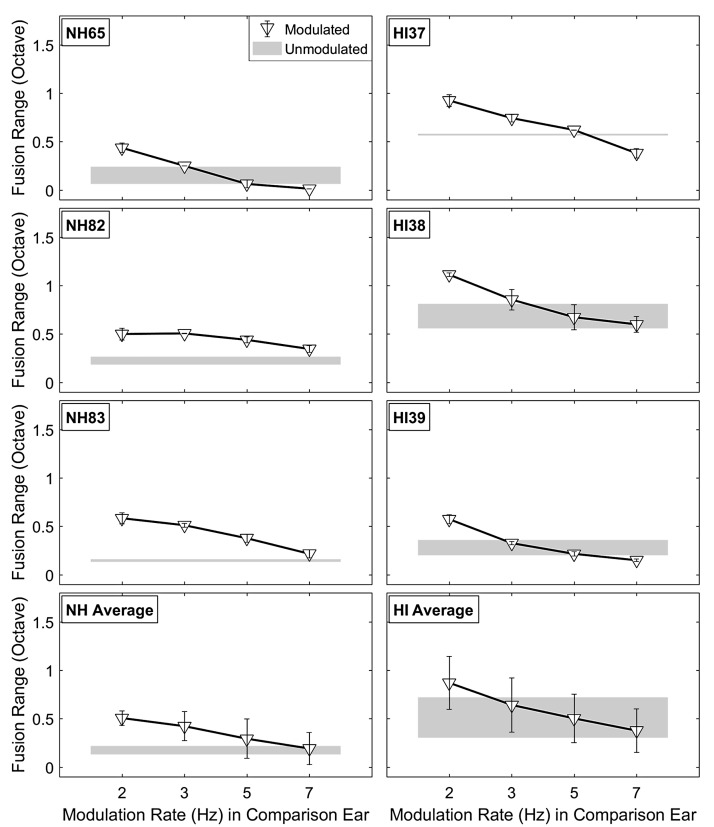
Effects of interaural AM rate differences on fusion range results for representative NH (left) and HA (right) subjects. The bottom panels show the averaged results for both subject groups. The upside-down triangle symbols indicate mean fusion ranges (in octave) with the fixed modulation rate at 2 Hz in the reference ear and 2-, 3-, 5-, and 7-Hz modulation rates in the comparison ear. As in Figure 6, the modulation depth is fixed at 60% for all conditions, and the shaded area represents fusion ranges (including standard deviations) in the unmodulated condition. HI = hearing impaired; NH = normal hearing.

To show the effects of interaural AM rate differences for the second incoherent AM condition, Figure 7 shows the individual and averaged fusion range results as a function of AM rate (2, 3, 5, and 7 Hz) in the comparison ear, paired with a fixed 2-Hz AM rate and 60% AM depth in the reference ear. The starting phases of the stimuli were randomized for each trial. In the same AM rate condition (2 Hz/2 Hz for reference/comparison ear), these incoherent phases resulted in fusion ranges between those observed in 0° (or 180°) and 90° phase difference conditions for most subjects. Overall, the results of the AM rate difference condition were similar to the interaural AM phase difference condition. The interaural AM rate difference led to narrower fusion ranges than those observed with coherent AM. The individual results showed reduced fusion ranges with different decreasing slopes for each subject.

Similar to the first incoherent AM condition, a significant main effect of interaural AM rate differences on fusion ranges was observed, *F*(3, 12) = 37.306, *p* < .0001, $\eta_{p}^{2}$^ ^= 0.903, RM-ANOVA with AM rate differences as within-subject factors and listener group as between-subject factor, but no main effect of listener group or interaction (*p* > .1 in both cases). Post hoc analyses using Bonferroni correction indicated that interaural AM rate differences led to significantly decreased fusion ranges compared with the coherent AM (2 Hz/2 Hz for reference/comparison ear) condition (*p* < .042, *p* < .027, and *p* < .012 for 2 Hz/3 Hz, 2 Hz/5 Hz, and 2 Hz/7 Hz for reference/comparison ear, respectively). Results from a separate RM-ANOVA with fusion center as the dependent variable showed no significant main effects of both interaural AM rate difference and listener group on the fusion-center shifts (*p* > .08 in both cases).

## Discussion

To our knowledge, this is the first study to systematically investigate how binaural pitch fusion can be influenced by temporal characteristics of the stimuli. The results show that coherent AM promotes binaural pitch fusion at low AM rates (2–8 Hz) and that incoherent AM influences binaural pitch fusion less strongly than coherent AM. The weaker effect of incoherent AM suggests an important role of temporal coherence in promoting fusion. Note that the effects of AM were significant for fusion range, but not for fusion-center shift. More important, these effects of AM cues on binaural pitch fusion show that fusion is not determined solely by peripheral processes that govern frequency resolution. Instead, the effects of AM suggest that binaural pitch fusion is mediated at least in part by central processes involved in auditory grouping.

Temporal coherence may provide a strong auditory grouping cue that increases fusion of dichotic spectral components, even when they are spectrally remote. Similar effects of coherent AM have been observed on dichotic grouping of spectral components in other auditory perception experiments. For example, comodulation masking release (CMR) is a perceptual phenomenon in which masked thresholds are decreased when the masker is amplitude-modulated (Hall, Haggard, & Fernandes, 1984). Here, the detectability of a tone signal masked by one noise band centered on the signal (i.e., on-signal band) can be improved by adding flanking noise bands that have the same temporal fluctuation as the on-signal band. Further, CMR still occurs when the comodulated flanking maskers are presented to the contralateral ear (Buss & Hall, 2008; Cohen & Schubert, 1987; Hall & Grose, 1990; Schooneveldt & Moore, 1987, 1989). This dichotic CMR phenomenon may occur because temporal coherence promotes perceptual grouping, or fusion, between the on-signal band and contralateral flanking masker bands across the ears and thus increases target detectability due to greater perceptual dissimilarity between the target and the fused masker. However, the frequency range over which dichotic CMR occurs in NH listeners is, on average, 1 octave below and 0.67 octaves above the 1-kHz target signal frequency (Cohen & Schubert, 1987), and broader than the fusion ranges observed with coherent AM in this study (−0.3 ∼ 0.2 octaves centered at 2 kHz). This discrepancy may be a consequence of different stimulus conditions between the current fusion study and the CMR studies: AM tones versus AM noise, and slow versus fast envelope fluctuations (2, 4, and 8 Hz vs. ∼ 15 Hz). Further study of fusion using nontone stimuli such as complex tones and narrowband noises could be helpful to better understand the mechanisms underlying dichotic CMR.

Another example of the effects of temporal coherence across frequency can be found in modulation detection interference (MDI) studies. MDI is the elevation in modulation detection threshold caused by spectrally distant, modulated maskers (Yost & Sheft, 1989). The amount of MDI is maximized when temporal coherence occurs between the signal and maskers and is gradually reduced with increasing separation between two modulation rates. Similar interference effects were also observed in the dichotic stimulation configuration, though the amount of interference was smaller than that in the monaural condition (Bacon & Opie, 1994; Mendoza, Hall, & Grose, 1995; Sheft & Yost, 1997). This effect may occur because increased fusion due to temporal coherence (comodulation) between the target and contralateral maskers hinders the perception of the attributes, such as AM, of each individual component. This dichotic MDI effect was maximized in NH listeners when the masker frequency was −0.33 octaves below and 0.33 octaves above the 993-Hz target frequency and minimized (or eliminated) at −1.14 and 0.74-octave spectral separation of the target and masker (Mendoza et al., 1995). Again, these frequency ranges for the effective MDI are much wider than the pitch fusion ranges observed in the current study for NH listeners, which may be due to differences in experimental conditions.

Variability in the effects of coherent and incoherent AM on fusion range was also observed in both subject groups. Both dichotic CMR and MDI studies (Bacon & Opie, 1994; Cohen & Schubert, 1987; Hall & Grose, 1990; Mendoza et al., 1995; Schooneveldt & Moore, 1987, 1989; Sheft & Yost, 1997) also showed individual variability in the amount of masking modulation in both NH and HI listeners. This may be attributable to the individual variability in the relative importance of coherent AM in perceptual grouping under dichotic conditions. In addition, the already broad fusion in HI listeners may explain why the effect of temporal coherence on both CMR and MDI was reduced in HI listeners compared with NH listeners.

Speech is subject to similar principles of perceptual grouping in various listening conditions. As observed in the study by Broadbent and Ladefoged (1957), different formants of a speech sound tend to be grouped together when they are coherently modulated. This grouping is emphasized even when two formants are perceptually competing under more complex conditions in dichotic presentation such as different fundamental frequencies and temporal asynchrony (Darwin, 1981). Moreover, Summerfield and Culling (1992) showed that AM incoherence cues such as different AM rates or phases can reduce the degree of grouping in concurrent vowel perception, similar to what was observed in this study for dichotic tones. Therefore, coherent AM may exacerbate inappropriate grouping of speech at the phoneme level, such as that seen in HI listeners with abnormally broad fusion, and promote inappropriate speech fusion (Cutting, 1976) and binaural interference (Reiss et al., 2016). Findings from the current study show that the likelihood of binaural fusion for spectrally distant components is promoted by adding temporal characteristics. Broadened fusion may be one underlying factor for reduced binaural benefits for speech perception in quiet and in noise in individuals with hearing loss.

## Conclusion

The current study focused on effects of AM on binaural pitch fusion in both NH subjects and HA users. In both groups, coherent AM promoted binaural pitch fusion at low AM rates (2–8 Hz) in the range known to contribute significantly to speech intelligibility. A small increment of AM depth had a significant effect on binaural pitch fusion. In addition, for both subject groups, incoherent AM with interaural AM rate and AM phase differences led to smaller increases in binaural pitch fusion, compared with the coherent AM conditions. Because speech sounds vary in both spectral and temporal domains, measurements of binaural pitch fusion with stimuli having both spectral and temporal characteristics may yield more realistic estimates of binaural fusion and provide a better understanding of how fusion affects binaural speech perception benefits for HI listeners.

## Authors' Note

Portions of this work were presented at the Meeting of the Association for Research in Otolaryngology, Baltimore, MD, February 2017.
